# A Saponification Method for Chlorophyll Removal from Microalgae Biomass as Oil Feedstock

**DOI:** 10.3390/md14090162

**Published:** 2016-09-07

**Authors:** Tao Li, Jin Xu, Hualian Wu, Guanghua Wang, Shikun Dai, Jiewei Fan, Hui He, Wenzhou Xiang

**Affiliations:** 1 CAS Key Laboratory of Tropical Marine Bio-Resources and Ecology (LMB-CAS), Guangdong Key Laboratory of Marine Materia Medica (LMMM-GD), South China Sea Institute of Oceanology, Chinese Academy of Sciences, Guangzhou 510301, China; taoli@scsio.ac.cn (T.L.); hlwu@scsio.ac.cn (H.W.); wgh@scsio.ac.cn (G.W.); deepseabio@163.com (S.D.); fanjiewei@scsio.ac.cn (J.F.); gz_heh@hotmail.com (H.H.); 2 Key Laboratory of Renewable Energy, Guangzhou Institute of Energy Conversion, Chinese Academy of Sciences, Guangzhou 510640, China; xujin@ms.giec.ac.cn

**Keywords:** microalgae oil, chlorophyll removal, saponification, *Scenedesmus*, sodium copper chlorophyllin

## Abstract

Microalgae oil is an optimal feedstock for nutraceutical, pharmaceutical and biodiesel production, but its high levels of chlorophyll limit its large-scale application. To date, few effective approaches have been developed to remove chlorophyll from microalgae oil. The main purpose of this study was to present a preprocessing method of algae oil feedstock (*Scenedesmus*) to remove chlorophyll by saponification. The results showed that 96% of chlorophyll in biomass was removed. High quality orange transparent oil could be extracted from the chlorophyll reduced biomass. Specifically, the proportion of neutral lipids and saturation levels of fatty acids increased, and the pigments composition became carotenoids-based. The critical parameters of chlorophyll reduced biodiesel conformed to the standards of the USA, China and EU. Sodium copper chlorophyllin could be prepared from the bleaching effluent. The results presented herein offer a useful pathway to improve the quality of microalgae oil and reduce the cost of microalgae biodiesel.

## 1. Introduction

Oleaginous microalgae can accumulate large amounts of storage oil (mainly triacylglycerol) under stressful environmental conditions [1]. Microalgae oil is considered an optimal feedstock for biodiesel production, and is also of great commercial value in nutraceutical, pharmaceutical, and health-care products [2]. However, liposoluble intracellular pigments are readily co-extracted by hexane into crude oil [3], which can have serious negative impacts on the down-stream processing and quality of oil [4].

Photosynthetic microalgae contain various intracellular pigments, such as chlorophyll (*a*, *b*, *c*, and *d*) and carotenoids (astaxanthin, lutein and beta-carotene), which can endow microalgae with green, orange and red color. Among these pigments, the most abundant is chlorophyll [5]. Chlorophyll is a magnesium-porphyrin compound that consists of a ring porphyrin (head) and a long hydrocarbon phytol (tail), both of which are bonded together by an ester bond [5]. Chlorophyll is water-insoluble, but can be easily dissolved in organic solvents such as ethanol, acetone, ether, and chloroform [5]. Aronoff (1960) recognized nine types of chlorophylls, among which the most common types are chlorophyll *a* (C_55_H_72_O_5_N_4_Mg, –CH_3_ as its functional group) and chlorophyll *b* (C_55_H_70_O_6_N_4_Mg, –CHO as its functional group) [6]. Chlorophyll is unstable in the presence of high light irradiance, acids, bases, and oxygen [7]. However, as long as its porphyrin ring structure is not destroyed, it retains its remarkable brown or green color. Although chlorophyll and its derivatives have many important biological functions, it should not be present in oil feedstock [4,8,9,10]. This is because chlorophyll makes the oil more susceptible to photo-oxidation, decreases the storage stability of oil, and causes an off-flavor [11]. Previous studies indicated that high concentration of chlorophyll in canola oil, which was widely used as biodiesel feedstock in the Europe Union (EU), led to the presence of low-quality oil with a dull and dark color [9,10]. More importantly, the presence of chlorophyll can obviously decrease the transesterification efficiency and combustion efficiency of biodiesel. This is also true of microalgae oil; therefore, chlorophyll removal should be as a key process in the commercial application of microalgae oil.

A number of studies have investigated chlorophyll removal from canola oil [12,13,14]. Conventional methods for chlorophyll removal include physical absorption, oxidation treatment and phosphoric acid degumming [9,13,14]. Baroi et al. (2013) reported that the catalyst improved the quality of the biodiesel by adsorbing chlorophyll from the feedstock (75.56% chlorophyll removal), during transesterification reaction [12]. Another study from Bahmaei et al. (2005) showed that concentrations of chlorophyll of up to 30 ppm could be reduced to amounts of less than 0.01 ppm by mixing the crude canola oil with a 0.4 wt % mixture of phosphoric and sulfuric acids (2:0.75, v/v) for 5 min at 50 °C [13]. Przybylski et al. (2005) reported that the total chlorophyll content of canola oil was reduced to less than 1 ppm after bleaching with activated bleaching clays [15]. Typically, the concentration of chlorophyll in crude canola oil is 13–30 ppm [8], which is much lower than the levels found in microalgae oil. Indeed, estimation of the level of chlorophyll in microalgae oil according to the following parameters: oil content (average 30% by dry weight (DW) [1], oil density (0.8 g·mL^−1^), and chlorophyll content (0.2%–1.5% DW) [16], and extraction efficiency of oil and chlorophyll (90%) indicates levels of crude microalgae oil of 5320–39,998 ppm, which was 117–3076 times greater than that in canola oil. There is little literature available regarding removal of chlorophyll from microalgae oil. It is not clear whether the above bleaching approaches for canola oil are suitable for microalgae oil. Sathish and Sims (2012) exhibited that a wet lipid extraction procedure was capable of removing most of the chlorophyll contamination of the algal lipid extract through precipitation [17]. Chen et al. (2012) showed that treatment with bleaching earth caused the content of chlorophyll and total carotenoids in *Scenedesmus* sp. to decrease from 4296.7 and 1918.9 ppm to 40.3 and 199.0 ppm, respectively [18]. However, these methods were not useful for large-scale application. That motivates us to conduct the study.

Chlorophyll can be saponified in the presence of sodium hydroxide, leading to the production of water-soluble chlorophyllin and phytol (C_55_H_72_O_5_N_4_Mg + 2NaOH = C_34_H_30_O_5_N_4_MgNa_2_ + 2CH_3_OH + C_20_H_39_OH) [19]. Based on the saponification reaction, chlorophyll can be easily separated from other liposoluble biochemical compounds. Accordingly, this saponification treatment is often used in the determination of carotenoids content [20]. Due to the similar absorption wavelength of chlorophyll and carotenoids at 440–490 nm, chlorophyll in biomass greatly interferes the determination of carotenoids. Yuan and Chen (1998) showed that chlorophyll in *Haematococcus* *pluvialis* must be removed by saponification treatment before the measurement of carotenoids content [21]. Granado et al. (2001) presented a fast, reliable and low-cost saponification protocol for analysis of carotenoids in vegetables [22]. These preprocessing methods give us a great enlightenment on chlorophyll removal from microalgae oil. It is generally known that both chlorophyll and oil can react with sodium hydroxide. In our previous study, crude microalgae oil was directly saponified to remove chlorophyll; however, a large proportion of oil was also lost (up to 90%). This may have occurred because microalgae oil completely exposed to sodium hydroxide, and was much easier to be saponified by sodium hydroxide. In consideration of the cellular complex constructions (e.g., thick cell wall, and oil body with being wrapped by other organelles), it is speculated that these cellular barriers may prevent the saponification reaction of storage oil. Based on the above hypothesis, the two-step bleaching approach, which includes saponification treatment of biomass in situ and oil extraction from the de-chlorophyll biomass, is presented in this study. This bleaching approach for microalgae oil is not reported, and is novel.

*Scenedesmus* sp. is an oleaginous microalga isolated by our laboratory that can accumulate high levels of total lipids (TL) (up to 55.9% DW) and secondary astaxanthin under nitrogen limitation. This microalga was confirmed to be culturable in an outdoor raceway system up to 250 m^2^, and its biomass productivity reached 15.0 g·m^−2^·day^−1^. Another important study for *Scenedesmus* sp. was conducted to domesticate it to grow in natural seawater. The domesticated *Scenedesmus* sp. could grow rapidly in 35‰ seawater and obtains over 7.0 g·L^−1^ biomass, which is more than that of freshwater (unpublished). However, high levels of chlorophyll have limited the application of *Scenedesmus* oil as a biodiesel feedstock. Therefore, this study was conducted to remove chlorophyll from *Scenedesmus* oil. Several traditional bleaching methods, including activated diatomite, oxidation treatment, phosphoric acid degumming, and gel silica column chromatography were applied to remove the chlorophyll from crude microalgae oil. The morphology, ultrastructure and biochemical composition of the chlorophyll reduced biomass, as well as the characteristics of the de-chlorophyll oil were analyzed in the study. The results presented herein provide a useful pathway to improve the quality of microalgae oil and reduce the cost of microalgae biodiesel.

## 2. Results

### 2.1. Traditional Bleaching Methods for Crude Scenedesmus Oil

**Figure 1:**
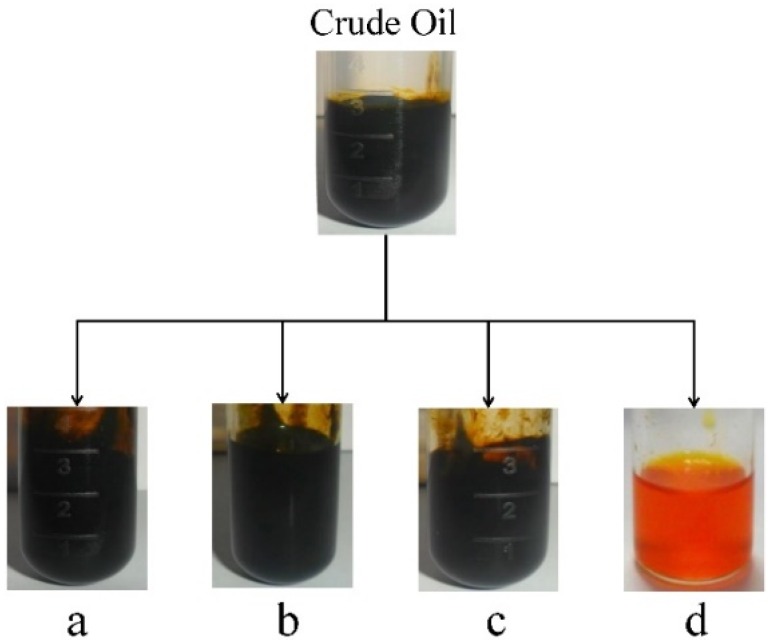
Traditional bleaching methods for the crude oil of *Scenedesmus* sp.: (**a**) activated diatomite at 80 °C for 2 h; (**b**) hydrogen peroxide at room temperature for 1 h; (**c**) 1% H_3_PO_4_ at 80 °C for 30 min; and (**d**) the gel column chromatogram.

Physical absorption, oxidation treatment, and phosphoric acid degumming have often been used for the refinement of vegetable oil and waste cooking oil. In this study, four methods: (a) activated diatomite at 80 °C for 2 h; (b) hydrogen peroxide at room temperature for 1 h; (c) 1% H_3_PO_4_ at 80 °C for 1 h; and (d) gel silica column chromatography (chloroform elution), were selected to remove chlorophyll from *Scenedesmus* oil. As shown in Figure 1, the color of the untreated crude oil was still black following treatment a, b and c, which was similar to the color of the original crude oil. Following treatment d, the crude oil became transparent orange. However, the gel silica column chromatography (treatment d) was very time-consuming and could not be easily used in large-scale production.

### 2.2. Selection of the Optimal Method for Chlorophyll Removal from the Dried Biomass

**Figure 2:**
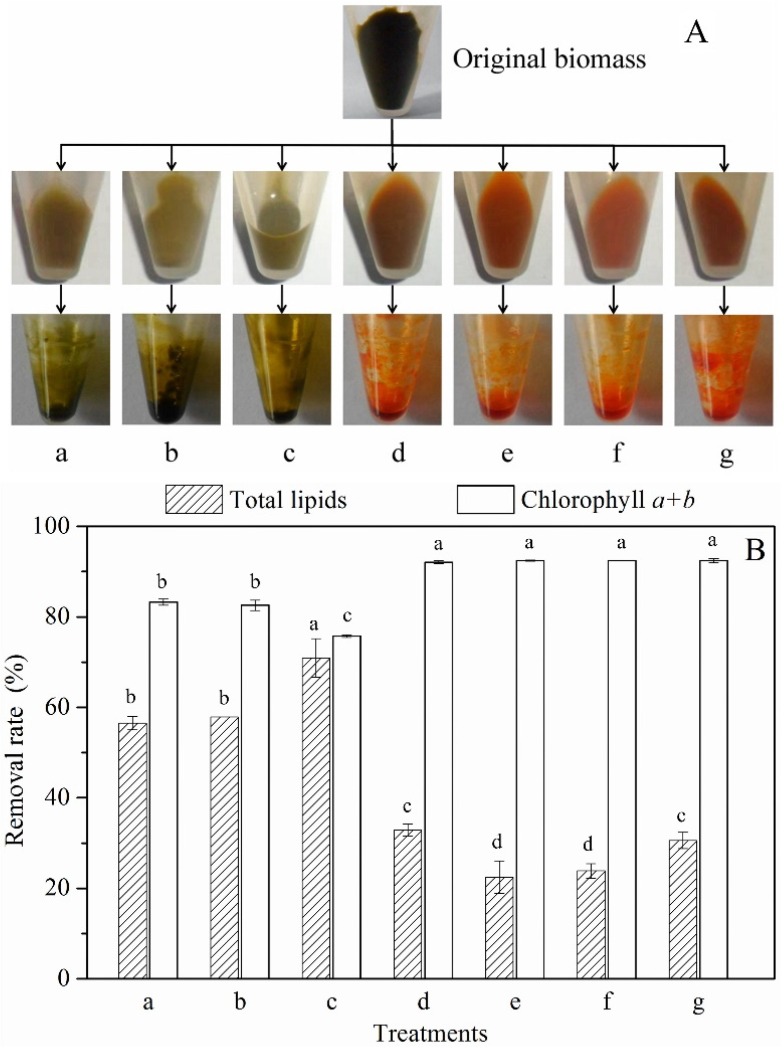
Selection of the optimum method for chlorophyll removal from the biomass: (**A**) treated biomass and the extracted crude oil; (**B**) the loss rate of chlorophyll (*a* + *b*) and total lipids. (**a**) 100% methanol at 70 °C for 1 h; (**b**) 100% ethanol 70 °C for 1 h; (**c**) 100% acetone at 70 °C for 1 h; (**d**) 1% NaOH at 70 °C for 1 h; (**e**) 100% methanol + 1% NaOH (1:4, v:v) at 70 °C for 1 h; (**f**) 100% ethanol + 1% NaOH (1:4, v:v) at 70 °C for 1 h; and (**g**) 100% acetone + 1% NaOH (1:4, v:v) at 70 °C for 1 h.

Seven methods were applied to remove chlorophyll from the dried biomass of *Scenedesmus* sp.: (a) 100% methanol at 70 °C for 1 h; (b) 100% ethanol at 70 °C for 1 h; (c) 100% acetone at 70 °C for 1 h; (d) 1% NaOH at 70 °C for 1 h; (e) 100% methanol + 1% NaOH (1:4, v:v) at 70 °C for 1 h; (f) 100% ethanol + 1% NaOH (1:4, v:v) at 70 °C for 1 h; and (g) 100% acetone + 1% NaOH (1:4, v:v) at 70 °C for 1 h. The treated biomass and extracted crude oil is shown in Figure 2A. The biomass showed a yellow-green color after treatment a, b, and c, but was orange following treatment d, e, f, and g. The oil extracted from the biomass treated using a, b, and c was black, but was bright orange color after extraction from biomass treated using d, e, f, and g.

The removal rate of chlorophyll and the loss rate of total lipids (TL) was measured as shown in Figure 2B. Following treatments a, b, and c, the removal rate of chlorophyll ranged from 75.2% to 82.5%. However, treatment d, e, f, and g resulted in chlorophyll removal rates of 92.0% to 92.3%. These results indicated that adding NaOH into the bleaching solution was beneficial for chlorophyll removal. During bleaching, there should be a low loss rate of TL. As shown in Figure 2B, treatment c produced the highest loss rate of TL (70.9%) (*p* < 0.01), while it was lowest in response to treatment e and f (22.5% and 23.9%, respectively). Based on comprehensive analysis (safety, chlorophyll removal rate and loss rate of TL), treatment f (ethanol-NaOH) was selected as the optimal condition for chlorophyll removal in our further studies.

### 2.3. Effects of the Chlorophyll Removal Process on Biochemical Composition of Biomass

**Figure 3:**
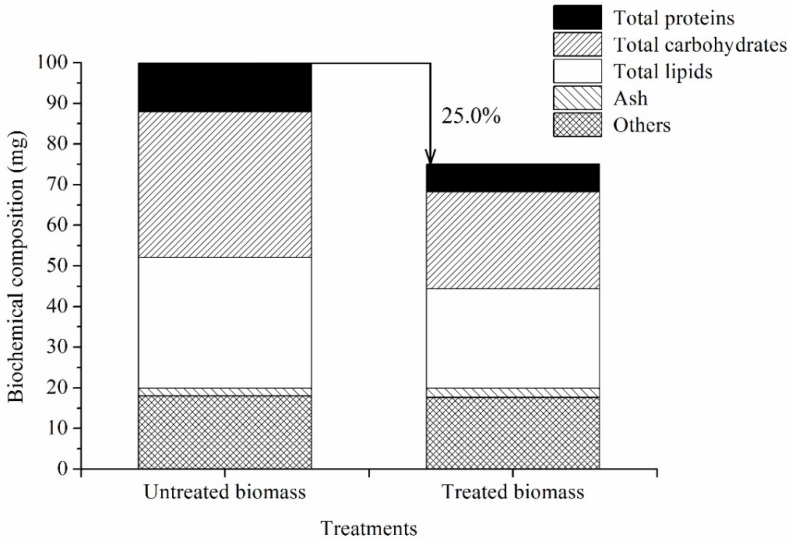
Effects of decoloring process on biochemical composition in *Scenedesmus* sp.

To determine the effects of treatment f (ethanol-NaOH) on the biochemical composition of *Scenedesmus* sp., the content of TL, total carbohydrates, total proteins and ash was measured. After treatment with ethanol-NaOH solution, the total weight of chlorophyll reduced biomass decreased 25.0% relative to the original biomass (Figure 3). The main contributors of biochemical substrates to the loss biomass were then analyzed. As shown in Figure 3, the content of TL, total carbohydrates, total proteins, ash and other compounds (mainly cell wall, water, DNA and RNA) was 32.2 mg, 35.9 mg, 12.1 mg, 1.9 mg, and 18.0 mg in 100 mg of the original biomass, but 24.5 mg, 23.8 mg, 6.8 mg, 2.3 mg, and 17.6 mg in 75 mg of the chlorophyll reduced biomass, respectively. The TL, total carbohydrates, total proteins, and other compounds decreased 23.8% (*p* < 0.01), 33.6% (*p* < 0.01), 43.9% (*p* < 0.01), and 2.2%, respectively, but ash increased 22.9% (*p* < 0.01). The proportion of TL in chlorophyll reduced biomass was 32.7% DW, which was 1.6% higher than that of the original biomass (*p* > 0.05).

### 2.4. Effects of the Chlorophyll Removal Process on Morphology and Ultrastructure of Cells

**Figure 4:**
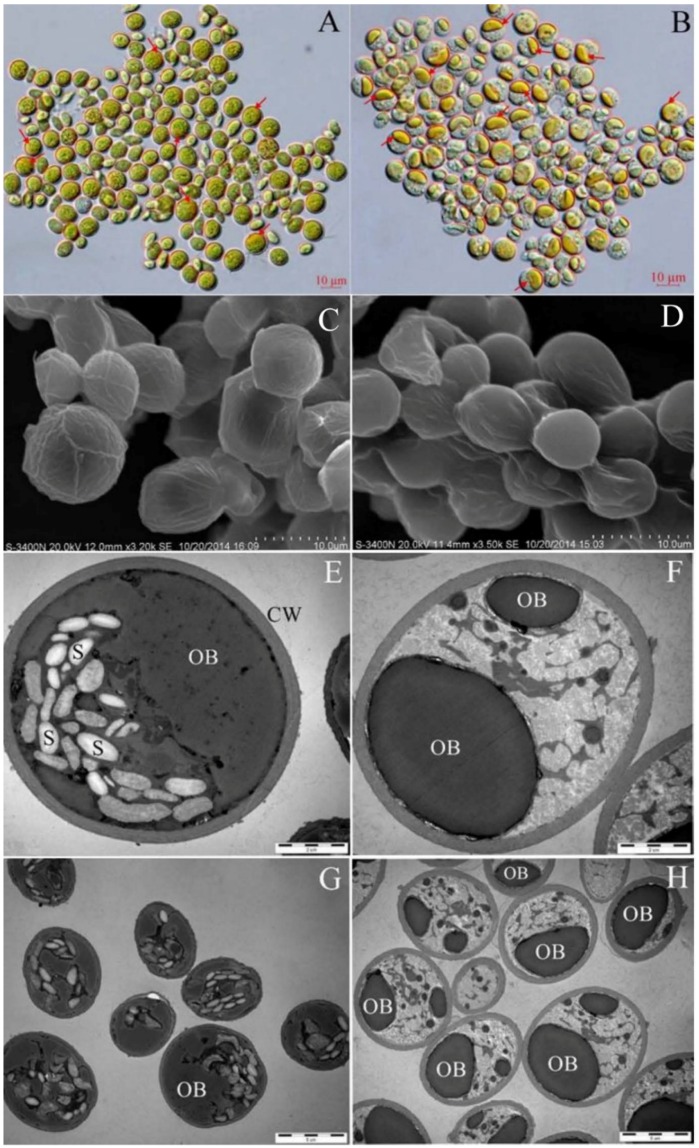
The morphology, surface feature and ultrastructure of *Scenedesmus* sp. cells: (**A**) the original cells under the optical microscope; (**B**) the de-chlorophyll cells under the optical microscope; (**C**) the original cells under the scanning electron microscope; (**D**) the de-chlorophyll cells under the scanning electron microscope; (**E**,**G**) the original cells under the transmission electron microscope; and (**F**,**H**) the de-chlorophyll cells under the transmission electron microscope. S: starch granule; OB: oil body; CW: cell wall; the red arrow: oil body.

The morphology of the de-chlorophyll cells was observed microscopically. As shown in Figure 4A,B, the de-chlorophyll cells remained spherical or ellipsoidal, but their intracellular structure and color changed significantly. Specifically, the de-chlorophyll cells became yellow. In the original cells, there were some small yellow-green cytoplasmic oil bodies inhomogeneously distributed in the cells that were mostly covered by chloroplasts. For the de-chlorophyll cells, the cytoplasmic oil bodies were gathered into a large one, and revealed.

The surface features of the original cells and the de-chlorophyll cells were examined using a scanning electron microscope (SEM) and are shown in Figure 4C,D. The de-chlorophyll cells remained spherical and ellipsoidal, but their cellular surface became smoother than that of the original cells. There were no obvious perforations in the cellular surface. These results indicated that ethanol-NaOH molecules might play an efficient role by diffusing into cells rather than perforating the membrane structure on the cellular surface.

The cellular ultrastructure was observed under a transmission electron microscope (TEM). As shown in Figure 4E–H, spindle starch granules and oil bodies were distributed in the original cells. However, ethanol-NaOH treatment caused the oil bodies to fuse into one large body, and the spindle starch granules disappeared completely (Figure 4F,H). Interestingly, a grey-white structure that may have been gelatinized starches appeared in the de-chlorophyll cells.

### 2.5. Fatty Acid (FA) Composition and Lipid Fractionation of De-Chlorophyll Oil

**Figure 5:**
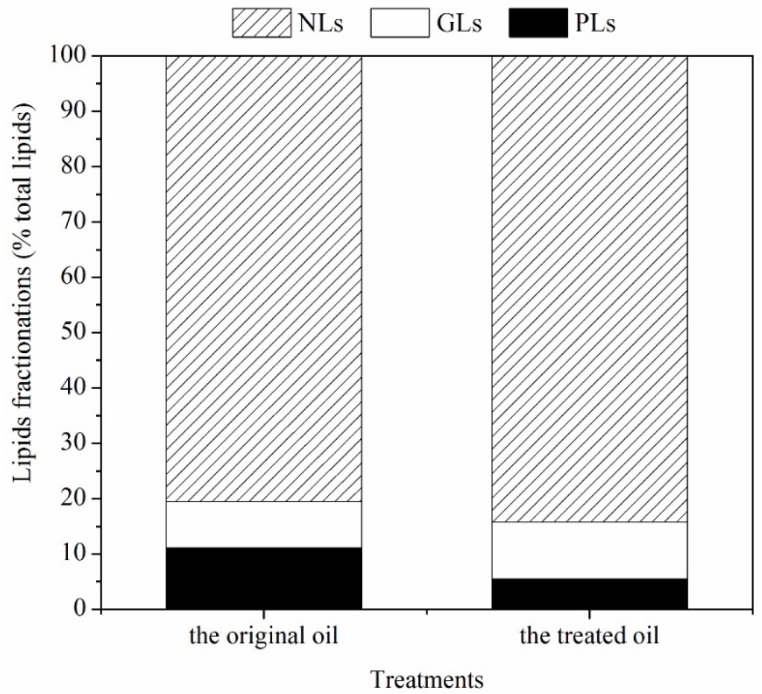
Lipid fractionation of the original oil and the treated oil.

TL could be further classified into neutral lipids (NLs), glycolipids (GLs), and phospholipids (PLs). Among these lipid fractionations, only NLs were easily transesterified into FA methyl ester for biodiesel production. For this reason, the proportions of NLs, GLs, and PLs in the crude oil were further analyzed (Figure 5). The proportion of NLs, GLs and PLs in untreated oil was 80.4% TL, 8.4% TL, and 11.1% TL, respectively. Upon treatment with ethanol-NaOH, the proportion of NLs, GLs and PLs in de-chlorophyll oil changed to 84.2% TL, 10.3% TL, and 5.5% TL, respectively. Overall, the proportion of NLs and GLs of de-chlorophyll oil increased by 3.8% (0.01 < *p* < 0.05) and 1.9% (*p* > 0.05) relative to the original oil, but the proportion of PLs decreased by 0.8% compared to the crude oil.

The FA composition of each lipid fractionation was analyzed using a gas chromatograph spectrometer with an FID detector. As shown in Table 1, the FA composition of untreated oil mainly included C16:0, C18:1, and C18:3 ω3 (over 10% total FA). The ethanol-NaOH treatment had almost no effect on the FA composition of NLs, but had a great effect on GLs and PLs (*p* < 0.01). The change in the proportion of saturated fatty acids (SFAs), mono-unsaturated fatty acids (MUFAs), and poly-unsaturated fatty acids (PUFAs) in NLs was less than 3% (*p* > 0.05). For GLs and PLs, the proportion of SFAs increased by 140.4% and 39.5% (*p* < 0.01), but the proportion of PUFAs decreased by 59.4% and 62.8% (*p* < 0.01), respectively. The greatest change of FA composition in GLs and PLs was C16:3, C18:3 ω3, and C20:2 (over 75%, *p* < 0.01).

### 2.6. Pigment Composition of De-Chlorophyll Oil

**Figure 6:**
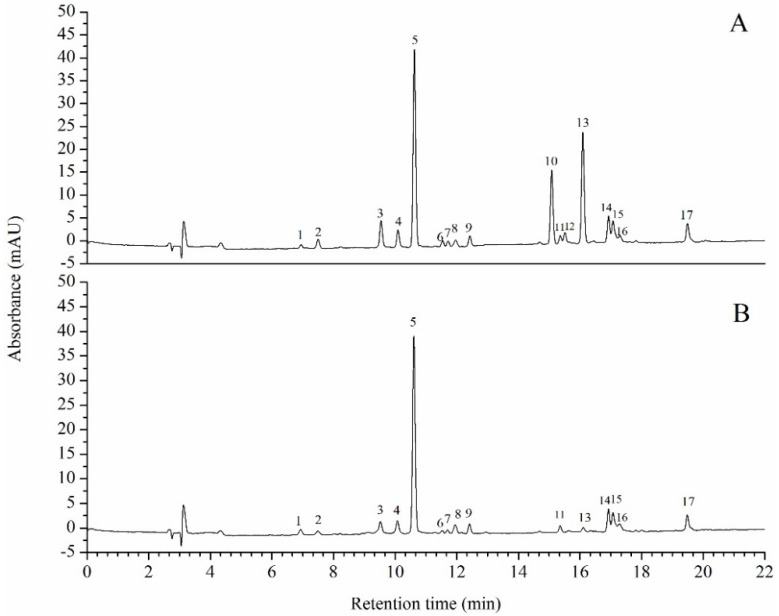
Pigments composition of the original oil (**A**) and the treated oil (**B**): (1) Violaxanthin; (2) Neoxanthin; (3) Canthaxanthin; (4) Unknown carotenoids; (5) Lutein; (6) Zeaxanthin; (7–8) Zeaxanthin ester; (9) Canthaxanthin isomer; (10) Chlorophyll *b*; (11) Chlorophyll *a* isomer; (12) Chlorophyll *b* isomer; (13) Chlorophyll *a*; (14–16) Astaxanthin ester; and (17) β-carotene.

The color of oil primarily originates from the pigments it contains. The pigments in the untreated oil mainly included violaxanthin, neoxanthin, lutein, zeaxanthin, canthaxanthin, chlorophyll *b*, chlorophyll *a*, astaxanthin, and β-carotene (Figure 6A). In Figure 6B, the peak of chlorophyll *a* and chlorophyll *b* of the de-chlorophyll oil almost disappeared. As shown in Table 2, the chlorophyll *a* and *b* decreased by 96.3% and 95.0% relative to the untreated oil (*p* < 0.01). Zeaxanthin showed the greatest decrease (58.1%), while lutein showed a minimal decrease (4.9%). Other high-value carotenoids such as β-carotene and astaxanthin decreased by 15%–23.6% (*p* < 0.01). The total carotenoids content of de-chlorophyll oil decreased by 23.3% relative to that of the original oil (Table 2). The absorption wavelength range of carotenoids ranged from 440 nm to 490 nm, which is in the orange region of the spectrum. Thus, the de-chlorophyll oil had an orange color.

### 2.7. Prediction of Biodiesel Quality Based on FA Composition

The FA composition of oil has an important effect on biodiesel quality. Specifically, the cetane number (CN), saponification value (SV), iodine value (IV), cold filter plugging point (CFPP), and long-chain saturated factor (LCSF) were predicted according to a theoretical model based on the FA composition. The quality parameters of biodiesel determined by the simulated calculation are listed in Table 3. The CN value of biodiesel based on de-chlorophyll oil increased 2.63% relative to the untreated oil (*p* < 0.01), indicating that the combustion speed of biodiesel improved. The IV indicates the degree of unsaturation of biodiesel. As shown in Table 3, the IV value of biodiesel derived from the de-chlorophyll oil decreased by 5.8% relative to that derived from the untreated oil. These results showed that the ethanol-NaOH treatment could increase the degree of saturation of biodiesel. The value of LCSF and CFPP was also related to the low temperature resistance of biodiesel. Few changes in LCSF and CFPP were observed after the bleaching process. The SV reflects the corrosivity of biodiesel. A high SV could increase the unburned carbon during diesel combustion, and reduce the combustibility of biodiesel. In this study, the ethanol-NaOH bleaching treatment had no impact on the SV of biodiesel.

### 2.8. Preparation of Sodium Copper Chlorophyllin (SCC)

**Figure 7:**
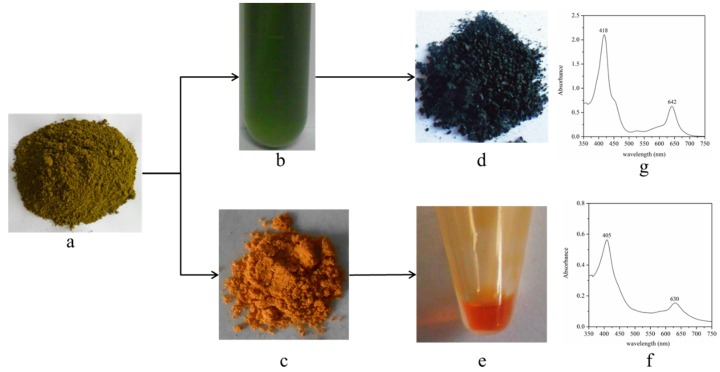
Preparation of sodium copper chlorophyllin: (**a**) the original biomass; (**b**) the bleaching effluent; (**c**) the chlorophyll reduced biomass; (**d**) the crude sample of SCC; (**e**) *Scenedesmus* oil; (**f**) absorption spectrum of the bleaching effluent; and (**g**) absorption spectrum of prepared SCC sample (SCC: sodium copper chlorophyllin).

SCC, a water-soluble commercial derivative of chlorophyll, has gained importance as a food colorant and dietary supplement with apparent chemopreventive activities. During bleaching, chlorophyll is saponified to generate water-soluble sodium magnesium chlorophyllin (SMC). In our study, the feasibility of preparing SCC from bleaching effluent containing SMC was studied. As shown in Figure 7, the yellow-green color biomass of *Scenedesmus* sp. (Figure 7a) was treated with ethanol-NaOH solution at 70 °C for 1 h. The orange chlorophyll reduced biomass (Figure 7c), the effluent containing SMC (Figure 7b), the deep green crude sample of SCC (Figure 7d), and the orange and transparent *Scenedesmus* oil (Figure 7e) acquired. Next, the quality of the prepared SCC sample was examined. The absorption spectrum of the prepared SCC sample is shown in Figure 7g. The maximum absorption peak was observed at 418 nm and 642 nm, which was the same as the standard of SCC. The features of the absorption spectrum differed between SMC and SCC. Specifically, the maximum absorption peak of SMC were 405 nm and 630 nm. The parameters related to the quality of SCC are shown in Table 4. For the prepared SCC sample, the value of E_1 cm_^1%^_405 nm_, which reflects the purity of SCC, was 59. The value of E_1 cm_^1%^_405 nm_ of the standard of SCC was 578. Based on these findings, the purity of the prepared SCC sample was only 10.2%, which is too low to meet the standards of China and the USA. However, the E_405 nm_/E_630 nm_ value and the pH of the prepared SCC was 3.63 and 8.25, which meets standard GB26406-2011 of China. The yield of SCC was 1.88 g/100 g biomass. Based on the chlorophyll content in the biomass of *Scenedesmus* sp. (0.48% DW), there was only 39.6% chlorophyll to transfer SCC. Moreover, the extraction efficiency of oil from the chlorophyll reduced biomass could reach 91.4% with 100% ethanol.

## 3. Discussion

Chlorophyll is an essential pigment for phototrophic microalgae [16]. Chlorophyll is almost completely co-extracted by hexane, after which it is mixed with the crude oil [3,4]. The presence of chlorophyll can produce serious negative effects on downstream processing of microalgae oil, such as transesterification and refinement [4]. According to our theoretical calculations, the chlorophyll concentration in crude microalgae oil was estimated to be 5320–39,998 ppm, which was 117–3076 times greater than that in canola oil (13–30 ppm) [8]. High concentrations of chlorophyll in oil are considered one of the key factors limiting its application to biodiesel [7,8,9,10,13,18].

In this study, several traditional bleaching methods: (a) activated diatomite; (b) hydrogen peroxide; (c) phosphoric acid degumming; and (d) gel silica column chromatography, were applied to remove chlorophyll from *Scenedesmus* oil. The results showed that activated diatomite, hydrogen peroxide and phosphoric acid degumming could not effectively eliminate chlorophyll from *Scenedesmus* oil (Figure 1). Chen et al. (2012) reported that treatment with bleaching earth caused the content of chlorophyll and total carotenoids in *Scenedesmus* sp. to decrease from 4296.7 and 1918.9 ppm to 40.3 and 199.0 ppm, respectively [18]. However, in this study, the effect of activated diatomite on chlorophyll removal was poor. This may have occurred because the chlorophyll concentration of the crude oil was too high, or chlorophyll was covalently coupled with protein so that it could not be separated by physical absorption. Gel silica column chromatography was time consuming and could not be easily used in large-scale production. Therefore, we considered four traditional bleaching methods were not effective method for chlorophyll removal.

Chlorophyll is a diester that can be saponified by alkali treatment, producing chlorophyllin [19]. To determine the effects of saponification treatment on chlorophyll removal, seven different treatments were investigated in this study. These treatments were divided into two groups: (1) 100% organic solvent without NaOH addition (treatment a, b and c); and (2) 20% organic solvent with NaOH addition (treatment d, e, f and g). Many previous studies have shown that the removal efficiency of pigments could reach more than 95% with 100% organic solvent extraction (e.g., methanol and acetone) [20]. There are two possible reasons that the 100% organic solvent group could not effectively dislodge pigments; namely, differences in microalgal species and differences in extraction conditions [23]. Moreover, 100% organic solvent treatment caused a great loss of TL (Figure 2B). This is because 100% organic solvent (methanol, acetone, and ethanol) has an excellent ability to dissolve liposoluble compounds [3]. All treatments with added NaOH resulted in recovery of transparent orange microalgae oil, chlorophyll removal efficiencies of up to 90%, and TL loss rates of less than 25% (Figure 2A,B). The above results demonstrated that the saponification treatment could remove chlorophyll indirectly from crude *Scenedesmus* oil.

Both chlorophyll and lipids (mainly triacylglycerol) can react with alkali bases such as sodium hydroxide [19]. Our results showed that chlorophyll removal based on saponification led to decreased TL in biomass. Interestingly, the decrease in chlorophyll (more than 90%) was greater than that of TL (lower than 25%). We speculated that chlorophyll might be saponified more easily than TL (mainly triacylglycerol). Chlorophyll and triacylglycerol had different free energy of saponification reaction. Unfortunately, the enthalpy and entropy values of chlorophyll and triacylglycerol were not found in existing chemical databases (Thermodynamic Databases of Thermo-Calc Software; Thermochemical Database of HSC Chemistry Software). If applied to large-scale production, the aforementioned decrease in TL could lead to great economic losses. By treatment, 96% of chlorophyll in crude oil is removed, and the high quality of orange transparent oil is obtained. Negatively, the saponification process causes a loss of at least 20% of the total lipids. We considers that the increased value of algae oil due to chlorophyll removal is far more than the loss of lipid. If the microalgae oil contains high concentration of chlorophyll, it can hardly be used in downstream products. In order to compensate the loss of the total lipids in the process of chlorophyll removal, several strategies are taken into account: (1) a layer of cotton-shaped substance floating on the surface of the saponified effluent is observed (Supplementary Figure S1). Most of the floating substance may be the sodium aliphatate (RCOONa). According to the previous literatures, RCOONa can be recovered and further used in the production of fatty acid methyl ester. Burja et al. (2007) performed a series of experiments to recover FA from sodium aliphatate that minimized loss and promoted the utilization efficiency of microalgal biomass [24]; (2) By optimization of temperature, time, concentration and volume ratio of sodium hydroxide, and ethanol concentration, the loss rate of total lipids keep less than 15% (unpublished); (3) We has made an attempt to prepare sodium copper chlorophyllin from de-chlorophyll effluent. The above results offer the available approaches to compensate the loss of oil.

Comparison of the morphological and ultrastructural changes in de-chlorophyll cells and the original cells could help us to understand the cytological mechanism of chlorophyll removal. Following treatment with heated ethanol-NaOH solution, the cells of *Scenedesmus* sp. retained their spherical or ellipsoidal shape, but their intracellular structure and color changed significantly (Figure 4). Additionally, starch granules disappeared, and the cytoplasmic scattered oil bodies formed one large compressed polymer of starches [25]. When heating, the starch granule expanded and disintegrated due to water absorption and gelatinization [26]. The gelatinized starch resulted in the formation of a white structure in the cells (Figure 4E–H). The starch granules were rather tough, but the oil body was soft [2,26]. For the untreated cells of *Scenedesmus* sp., the tough starch granules spread in the cell and formed some intracellular space barriers (Figure 4E–H). The soft oil body was separated into several discontinuous segments by tough starch granules. When the starch granules were gelatinized in hot water, the intracellular space barriers disappeared. Owing to the strong surface tension of oil, the scattered oil bodies tended to assemble one large spherical structure. No obvious perforation was observed in the cellular surface after treatment with ethanol-NaOH solution, indicating that sodium hydroxide might have directly permeated into the cells to react with chlorophyll rather than damaging the surface structure of the cells. The removal process of chlorophyll likely occurred as follows: (1) sodium hydroxide permeated into the cell and reacted with chlorophyll to generate water-soluble SMC; (2) the generated SMC molecules diffused outside the cell; and (3) some of the lipid molecules (NLs, GLs and PLs) were saponified and generated water-soluble sodium aliphatate that could be brought outside the cell, and most of the water-insoluble lipids molecules (NLs, GLs and PLs) were left in the cell (Figure 4). The ethanol in saponification solution could contribute to the solubilization of chlorophyll.

The major biochemical composition of microalgae included lipid, carbohydrate, and protein [27]. In this study, saponification led to decreases in the major biochemical compounds of varying degrees (Figure 3). The excellent solubility in heat alkaline solution was the major reason for the decrease in protein. Rausch et al. (1981) extracted soluble proteins by using 0.5 N NaOH [28]. In general, Scenedesmaceae contained two types of starch, amylose and amylopectin [25]. Kobayashi et al. (1974) reported that the amylose and amylopectin content of *Scenedesmus basilensis* was 22% and 78%, respectively [25]. Amylose can be dissolved in hot water to form colloidal solution; however, amylopectin swelled and gelatinized in hot water [26]. The solubility of amylopectin in water also depends on pH. According to Kobayashi, the loss of carbohydrate mainly originates from amylose. Glyceride reduction occurred owing to the lipid saponification reaction [7]. The lost biochemical substances to the bleaching effluent, which exerted a bad influence on the preparation of SCC.

FA composition and lipid fractionation has an important influence on the quality of oil and biodiesel [29]. NLs are more suitable as biodiesel feedstock than polar lipids (GLs and PLs) [1]. Garcia (2013) reported that the high proportion of polar lipids could impact oil transesterification [4]. Our results showed that the saponification treatment, which decreased the proportion of polar lipids but increased the proportion of NLs, could improve the oil quality. In the traditional process of oil refinement, caustic refining (saponification) has been used to remove impurities from neutral oil, such as free FA, pigments, protein, particles, and PLs and GLs to improve the quality of oil [4,30]. The FA composition of the de-chlorophyll oil was also changed greatly. The proportion of PUFAs decreased significantly relative to SFAs and MUFAs because most PUFAs are located in polar lipids (cell membrane and chloroplast membrane), which were highly saponified in this bleaching process. The change in FA composition impacts the quality of biodiesel (CN, LCSF, CFPP, IV, and SV) [29]. CN is related to the spontaneous combustion of biodiesel in diesel engines, while LCSF and CFPP are related to low temperature resistance of biodiesel [29], IV represents the degree of unsaturation of oil [31] and SV is related to internal engine deposits and nozzle coking [31]. The value of CN of biodiesel derived from *Scenedesmus* oil was 54.7, which conformed to the standards of the USA (ASTM6751), China (BD100), and the EU (EN14214). Based on the LCSF and CFPP, the low temperature resistance of chlorophyll-reduced biodiesel showed no obvious changes. The decreasing IV indicated that the stability of biodiesel was promoted. In our study, the value of SV was 195, which conformed to the standards of the USA (ASTM6751), China (BD100) and the EU (EN14214). Based on these findings, saponification treatment could enhance the quality of oil and make it more suitable as a biodiesel feedstock.

Microalgae contains many types of photosynthetic pigments. Our results showed that *Scenedesmus* sp. contained violaxanthin, neoxanthin, lutein, zeaxanthin, canthaxanthin, chlorophyll *b*, chlorophyll *a*, astaxanthin, and β-carotene. The pigment composition of *Scenedesmus* sp. was similar to that of previous studies [32]. The HPLC data and color of the extracted oil indicated that the chlorophyll *a* and chlorophyll *b* were almost completely wiped out. Several secondary carotenoids that were present in the form of fatty acyl mono- or diesters (e.g., zeaxanthin and astaxanthin) decreased greatly. These fatty acyl mono- or diesters of pigments could be saponified and decomposed [32]. Although many studies of microalgae oil extraction have been conducted to date, there is little literature available regarding removal of chlorophyll from microalgae oil [18]. In this study, a transparent orange *Scenedesmus* oil was obtained. Red oil containing astaxanthin has been extracted from *Haematococcus pluvialis* and widely used as the addition agent for nutraceutical and aquaculture industries. In addition to being useful as biodiesel feedstock, the transparent orange oil from *Scenedesmus* sp. might have a potential application in edible oil, nourishment, and cosmetics.

Water-soluble SCC has been widely applied as a food colorant and dietary supplement with apparent chemopreventive activities [19]. Following saponification treatment, chlorophyll was converted into SMC, which could be dissolved in the effluent. If de-chlorophyll effluent was discarded, it could cause environmental pollution and lead to the loss of high-value compounds. In this study, we attempted to prepare SCC from de-chlorophyll effluent based on an existing method [33]. The results showed that the purity of the prepared SCC was too low to meet the standards specified by GB26406-2011 and the FDA (Food and Drug Administration, Sec. 73.125). Shi et al. (2009) reported that the impurities (e.g., protein, salt and starch) could impact the process of SCC preparation and its purity [34]. At present, commercial SCC is primarily made from silkworm excrement in China [34]. Because the yield of silkworm excrement is low, the price of SCC reached 260–300 USD/kg on the market (Shanghai Herbary Biotechnology Co., Ltd., Shanghai, China). The preparation of SCC from the de-chlorophyll effluent of microalgae might provide a potential pathway to reduce the cost of SCC and microalgae biodiesel. In the future, we will optimize the process of SCC preparation to improve its purity and yield.

Taken together, the presented method for chlorophyll removal based on saponification was feasible for *Scenedesmus* sp. What is most important is that, for the art of our above technology, the whole process, including chlorophyll removal and lipids extraction, is conducted by using ethanol as the sole organic solvent, which is easily to scale up and industrialized with lower cost and high safety. However, this research has also led to several questions that require further investigation. Specifically, it is not clear if the saponification treatment for chlorophyll removal is suitable for different microalgae species or *Scenedesmus* sp. during other cultivation periods. Additionally, the kinetic characteristics of the saponification reaction of chlorophyll and glycerolipids should be evaluated. We considered the best bleaching principle to be identification of a balance point between the loss rate of TL and removal rate of chlorophyll by optimizing bleaching conditions. The proposed approach would make a greatly valuable contribution on the technology of comprehensive and whole utilization of microalgal biomass for production of oil and other valuable products.

## 4. Materials and Methods

### 4.1. Microorganisms and Culture Conditions

*Scenedesmus* sp. was isolated from a subtropical lake in Guangdong province of the southern China. The culture was grown in Ø 6.0 cm × 60 cm glass column with modified BG-11 medium which contained 5.8 mM NaNO_3_ (17.6 mM NaNO_3_ in standard BG-11 medium), 0.175 mM K_2_HPO_4_·3H_2_O, 0.304 mM MgSO_4_·7H_2_O, 0.245 mM CaCl_2_·2H_2_O, 0.241 mM NaCO_3_, 11.7 μM FeCl_3_·6H_2_O, 31.1 μM Citric acid, 11.7 μM EDTANa_2_·2H_2_O, 46.1 μM H_3_BO_3_, 9.15 μM MnCl_2_·4H_2_O, 0.77 μM ZnSO_4_·7H_2_O, 1.62 μM, Na_2_MoO_4_·2H_2_O, 9.15 μM Co(NO_3_)_2_·6H_2_O, 0.32 μM CuSO_4_·5H_2_O. Illumination was provided by a bank of fluorescent lamps on one side of the photobioreactors at 350 μmol photons m^−2^·s^−1^. The growth temperature was kept at 25 ± 1 °C. The carbon source and agitation were supplied by bubbling CO_2_-enriched compressed air (1% CO_2_, v/v). After 18 days of cultivation, the culture was harvested by using a tubular bowl centrifuge (GQ-45, 28,000 rpm). The biomass sludge were freeze-dried in a freezer dryer (FD-1-50, BIOCOOL, Beijing, China) and stored in −20 °C.

### 4.2. Experimental Design

Four bleaching methods were applied to remove chlorophyll from the *Scenedesmus* oil: (a) activated diatomite at 80 °C for 2 h; (b) hydrogen peroxide at room temperature for 1 h; (c) 1% H_3_PO_4_ at 80 °C for 30 min; (d) column chromatography.

Additionally, an optimal method for chlorophyll removal from *Scenedesmus* biomass was selected from seven different treatments: (a) 100% methanol at 70 °C for 1 h; (b) 100% ethanol at 70 °C for 1 h; (c) 100% acetone at 70 °C for 1; (d) 1% NaOH at 70 °C for 1 h; (e) 100% methanol + 1% NaOH (1:4, v:v) at 70 °C for 1 h; (f) 100% ethanol + 1% NaOH (1:4, v:v) at 70 °C for 1 h; (g) 100% acetone + 1% NaOH (1:4, v:v) at 70 °C for 1 h. Briefly, freeze-dried biomass (70 mg) was put into 10 mL glass centrifuge tubes containing 7 mL of treatment solution. Each treatment had three experimental replicates (*n* = 3). After evaluation, the optimal treatment was selected for further study.

Finally, we attempted to prepare SCC from the bleaching effluent, and extracted oil from the chlorophyll reduced biomass. Moreover, the purity of the prepared SCC sample was measured based on the standard of China and the United States.

### 4.3. Morphological and Ultrastructural Observation

Morphology was observed under light microscope Olympus BX41 (Olympus, Tokyo, Japan). The surface features of cells were investigated by SEM (S-3400N Hitachi, Tokyo, Japan) (accelerating voltage 20 KV and 30 KV, working distance 11,500 lm and emission current 77,700 nA). Cells were coated with a thin layer of gold, mounted on a copper stab using a double stick carbon tape and surfaces were made conductive using gold coatings. The ultrastructural observation of cells was conducted using a TEM (Tecnai 10, FEI, Hillsboro, OR, USA). The specimen preparation of TEM was the following: the cells were concentrated by centrifugation at low speed (2000 rpm), the pellets were picked using toothpick and transferred into a small bottle. The small cell pellets were pre-fixed in 2% glutaraldehyde in phosphate buffer solution (PBS) at 4 °C, pH 7.2 for 2 h, and then rinsed three times with the above buffer. Subsequently, the small cell pellets were post-fixed with 1% osmium tetroxide in PBS at 4 °C for 2 h, rinsed three time with PBS and dehydrated by a series of mixture of acetone and distilled water (30%, 50%, 70%, 90%, 95%, and 100%) at room temperature for 30 min, respectively. Finally, the dehydrated cell pellets were infiltrated with mixture of acetone and Epon 812 resin (1:1) for 3 h, then moved to mixture of acetone and Epon 812 resin (1:3) for 3 h and finally transferred to 100% resin overnight. The specimens were then solidified at 45 °C for 12 h and 60 °C for 24 h, respectively. The embedding blocks were sectioned with diamond knives. The ultra-thin sections of specimens were stained with uranyl acetate and lead citrate each for 30 min and observed under TEM [35].

### 4.4. Removal Efficiency of Chlorophyll in the Dried Biomass

The TL of the original biomass and treated biomass were extracted via a modified Khozin–Goldberg’s method, dried under N_2_ flow, and then dissolved in 100% methanol. The chlorophyll concentrations of the methanol solutions were detected and calculated according to the following equation:

Chl. (*a* + *b*) = (1.44 × A_665.2_ − 24.93 × A_652.4_) × V (1)

where Chl. (*a* + *b*) is the chlorophyll concentration; A_665.2_ and A_652.4_ are the absorbance at 665.2 nm and 652.4 nm, respectively; and V is the volume of methanol [36].

The removal efficiency of chlorophyll was calculated according to the following equation:

Removal efficiency of chlorophyll (%) = Chl (*a* + *b*)_1_/Chl (*a* + *b*)_0_ (2)

where Chl (*a* + *b*)_1_ is the chlorophyll concentration of the total lipids extracted from the treated biomass; and Chl (*a* + *b*)_0_ is the chlorophyll concentration of the total lipids extracted from the original biomass.

### 4.5. Biochemical Composition Determination

Freeze-dried biomass (100 mg) was used for the determination of biochemical composition including TL, total carbohydrates and total proteins. The modified Khozin–Goldberg’s method was applied to determinate TL content [37,38]. Free lipids residual biomass was used to measure the content of total carbohydrates and total proteins. Free lipids residue (10 mg) was hydrolyzed with 0.5 N H_2_SO_4_ at 80 °C for 1 h. The total carbohydrates content was measured by the phenol-sulfuric acid method [39]. The Kieldahl method was used to determine the content of total proteins. The biomass was place in a muffle furnace at 550 °C for 5 h for the determination of ash content.

### 4.6. Total Lipids Extraction from the Chlorophyll Reduced Biomass by 100% Ethanol

Freeze-dried (70 mg) and chlorophyll reduced biomass (70 mg) was put into 10 mL glass centrifuge tubes containing 7 mL of 100% ethanol at 60 °C for 1 h. The ethanol extract was mixed with hexane and water to form a ratio of 1:2:1 (v/v/v). The upper lipid-containing phase was collected, and dried at N_2_ flow.

### 4.7. Lipid Classification and FA Composition

TL were classified using the method of column chromatogram as described by Li et al. (2013) [37]. The individual lipid fractionations (NLs, GLs and PLs) were transmethylated with 2% H_2_SO_4_ in a methanol:toluene mixture (90:10, v/v) at 80 °C for 1.5 h, respectively. Heptadecanoic acid was added as an internal standard. FA methyl esters were analyzed by using a gas chromatograph-mass spectrometer equipped with a 100 m fused silica capillary column SP-2560. The temperature of the injection port was 260 °C. The column was temperature-programmed from 140 °C (a hold of 5 min) to 240 °C at 4 °C/min (a hold of 20 min). The carrier gas was high-purity nitrogen and the flow rate was 1.2 mL/min [38].

### 4.8. Analysis of Pigments Composition and Content in Microalgae Oil

The extracted TL of the original biomass and treated biomass was dissolved in 100% methanol. The concentration of total carotenoids was measured by spectrophotometry as described by Lichtenthaler (1987).

*C*_a_ = 16.72 × A_665.2_ − 9.16 × A_652.4_ *C*_b_ = 34.09 × A_652.4_ − 15.28 × A_665.2_ Total carotenoids = (1000 × A_470_ − 1.63 × *C*_a_ − 104.96 × *C*_b_)/221 (3)

where *C*_a_ and *C*_b_ are the concentrations of chlorophyll *a* and chlorophyll *b* (mg·L^−1^), respectively and A_665.2_, A_652.4_, and A_470_ are the absorbance of the lipids solution at 665.2 nm, 652.4 nm and 470 nm, respectively.

### 4.9. The Implications for Biodiesel Quality

The CN, SV, IV, CFPP, and LCSF was predicted according to the existing calculation system for biodiesel quality.

Based on the calculation model described by Stansell et al. (2012), CN of biodiesel was calculated following Equation (4) [29].
SFAs: CN = −107.71 + 31.126*n* − 2.042*n*^2^ + 0.0499*n*^3^        (4)MUFAs: CN = 109 − 9.292*n* + 0.354*n*^2^PUFAs: CN = −21.157 + (7.965 − 1.785db + 0.235db^2^)*n* − 0.099*n*^2^CN of biodiesel: CN = 1.068∑(CN*_i_* × *m_i_*) − 6.747 where *n* is the number of carbon of FA; db is the number of double bond of FA; CN*_i_* is the value of CN of different FA; and *m_i_* is the percentages of FA.

According to Zhou et al. (2013), the value of SV, IV, LCSF, and CFPP was predicted following Equations (5)–(8) [31].

(5) $$\mathrm{IV}=\frac{\sum \left(254\times \mathrm{db}\times m_{i}\right)}{M}\;$$

(6) $$\mathrm{SV}=\frac{\sum (560\times m_{i})}{M}\times 3$$

LCSF = (0.1 × C16) + (0.5 × C18) + (1 × C20) + (1.5 × C22) + (2 × C24)(7)

CFPP = 3.1417 × LCSF − 16.477(8)

where db is the number of double bond of FA; M is molecular weight of FA; *m_i_* is the percentages of FA; and C16, C18, C20, C22, and C24 are the percentages of FA.

### 4.10. Preparation of SCC and Its Quality Detection

The effluent containing SMC was collected and allowed to stand overnight. HCl (1:1, v:v) was then added into the effluent to adjust the pH to 2–3. 2–3.5 mL of 10% CuSO_4_ was then mixed with the effluent, after which the mixture was put into a water bath at 60 °C for 1 h. The copper chlorophyllin acid was then allowed to precipitate out as sediment, which was subsequently dissolved in 95% ethanol and adjusted to pH 11. About 10 min later, the flocculent of SCC was precipitated out. The sample of SCC was then collected by centrifugation, after which it was dried in an oven at 80 °C for 2 h [33].

Next, prepared SCC (10 mg) was dissolved into 100 mL H_2_O (0.01% SCC solution), after which 1 mL of the solution was transferred to 9 mL H_2_O to give 0.001% SCC solution.

The absorbance of 0.001% SCC solution at 405 nm and 630 nm was measured in a spectrophotometer. Equations (9)–(12) were subsequently used to determine the E_1 cm_^1%^_405 nm_, E_405 nm_/E_630 nm_, Yield (per 100 g biomass) and Purity (%).

E_1 cm_^1%^_405 nm_ = A_405 nm_ × 1000 (9)

E_405_/E_630_ = A_405 nm_/A_630 nm_ (10)

Purity (%) = E_1 cm_^1%^_405 nm_/578 (11)

Yield (per 100 g biomass) = m_1_/m_2_ (12)

where A_405 nm_ and A_630 nm_ are the absorbance of 0.001% of SCC solution at 405 nm and 630 nm, respectively; E_1 cm_^1%^_405 nm_ represents the extinction coefficient, and 578 is E_1 cm_^1%^_405 nm_ of SCC standard. The parameter of m_2_ was the weight of microalgae biomass and m_1_ was the weight of prepared SCC.

### 4.11. Statistical Analysis

Figures and tables show the means and standard deviations of three experimental replicates and three technical replicates. The data were analyzed statistically using analysis of variance (ANOVA). Multiple comparison between the groups was performed using S-N-K method. The difference between sample means was analyzed using the least significant difference (LSD) at a probability level of 0.05.

## 5. Conclusions

For *Scenedesmus* sp., the removal of chlorophyll in biomass by saponification treatment followed by the extraction of oil from the de-chlorophyll biomass was feasible and led to recovery of high quality, transparent orange *Scenedesmus* oil. SCC could be prepared from the bleaching effluent, but its purity was low. The predicted critical parameters of chlorophyll reduced biodiesel conformed to the standards of the USA, China and the EU. Overall, the results presented herein provide a useful pathway to improve the quality of microalgae oil and reduce the cost of microalgae biodiesel. The proposed approach will also make a valuable contribution to the comprehensive and complete utilization of microalgal biomass for production of oil and other valuable products.

## Figures and Tables

**Table 1:** Fatty acid composition of the original oil and the de-chlorophyll oil.

	NLs ^2^		GLs ^3^		PLs ^4^	
	Untreated Oil (% TFA ^1^)	De-Chlorophyll Oil (% TFA)	Untreated Oil (% TFA)	De-Chlorophyll Oil (% TFA)	Untreated Oil (% TFA)	De-Chlorophyll Oil (% TFA)
C16:0	29.7 ± 0.1	27.5 ± 0.1	18.4 ± 0.1	32.9 ± 0.2	42.0 ± 0.4	37.8 ± 0.2
C16:1	1.9 ± 0.0	1.6 ± 0.0	1.3 ± 0.0	0.7 ± 0.0	1.5 ± 0.0	1.2 ± 0.0
C16:3	2.8 ± 0.0	2.5 ± 0.0	5.2 ± 0.0	0.7 ± 0.0	2.4 ± 0.0	0.6 ± 0.0
C18:0	3.9 ± 0.0	4.7 ± 0.0	4.4 ± 0.0	20.2 ± 0.0	2.2 ± 0.0	21.2 ± 0.1
C18:1	38.9 ± 0.2	38.2 ± 0.4	37.5 ± 0.4	28.5 ± 0.1	14.2 ± 0.1	22.3 ± 0.2
C18:2	8.7 ± 0.0	9.5 ± 0.1	8.2 ± 0.1	6.9 ± 0.0	10.3 ± 0.0	5.7 ± 0.0
C18:3ω6	1.2 ± 0.0	1.8 ± 0.0	0.2 ± 0.0	2.3 ± 0.0	0.2 ± 0.0	3.2 ± 0.0
C18:3ω3	11.4 ± 0.1	12.9 ± 0.1	24.6 ± 0.2	5.5 ± 0.0	23.1 ± 0.0	5.2 ± 0.0
C20:0	0.3 ± 0.0	0.2 ± 0.0	0.2 ± 0.0	2.2 ± 0.0	0.1 ± 0.0	2.6 ± 0.0
C20:2	1.2 ± 0.0	1.3 ± 0.0	*n.d.* ^8^	*n.d.* ^8^	4.0 ± 0.0	0.2 ± 0.0
SFAs ^5^	33.8 ± 0.1	32.3 ± 0.1	23.0 ± 0.1	55.3 ± 0.1	44.3 ± 0.3	61.6 ± 0.2
MUFAs ^6^	40.8 ± 0.1	39.8 ± 0.2	38.7 ± 0.2	29.2 ± 0.1	15.7 ± 0.1	23.5 ± 0.1
PUFAs ^7^	25.4 ± 0.1	27.9 ± 0.1	38.2 ± 0.1	15.5 ± 0.0	40.0 ± 0.0	14.9 ± 0.0

^1^ TFA: total fatty acids; ^2^ NLs: neutral lipids; ^3^ GLs: glycolipids; ^4^ PLs: phospholipids; ^5^ SFAs: saturated fatty acids; ^6^ MUFAs: mono-unsaturated fatty acids; ^7^ PUFAs: ploy-unsaturated fatty acids; ^8^ *n.d.*: not detected.

**Table 2:** Pigments composition of the original oil and the treated oil.

No.	Name of Pigment	Decreased Ratio (%)
1	Violaxanthin	0
2	Neoxanthin	54.3 ± 1.0
3	Canthaxanthin	48.0 ± 0.3
4	Unknown carotenoids	21.9 ± 0.4
5	Lutein	4.9 ± 0.1
6	Zeaxanthin	58.1 ± 0.7
7	Zeaxanthin ester	50.3 ± 0.2
8	Zeaxanthin ester	0
9	Canthaxanthin isomer	17.6 ± 0.5
10	Chlorophyll *b*	95.0 ± 0.8
11	Chlorophyll *a* isomer	7.7 ± 0.4
12	Chlorophyll *b* isomer	98.5 ± 0.2
13	Chlorophyll *a*	96.3 ± 1.2
14	Astaxanthin ester	23.6 ± 0.1
15	Astaxanthin ester	15.0 ± 0.4
16	Astaxanthin ester	19.8 ± 0.1
17	β-Carotene	23.8 ± 0.5
Chlorophyll (*a* + *b*)		96.0 ± 0.6
Total carotenoids		23.3 ± 0.4

**Table 3:** Quality properties of biodiesel derived from original oil and de-chlorophyll oil.

	C16 (% TFA ^6^)	C18 (% TFA ^6^)	C20 (% TFA ^6^)	CN ^1^	LCSF ^2^	CFPP ^3^	IV ^4^	SV ^5^
Original oil	34.8 ± 0.8	63.4 ± 1.3	1.7 ± 0.1	53.3 ± 0.1	0.37 ± 0.02	−15.3 ± 0.0	97.4 ± 0.2	195.9 ± 0.5
De-chlorophyll oil	32.3 ± 0.6	66.1 ± 2.0	1.6 ± 0.0	54.7 ± 0.2	0.38 ± 0.01	−15.3 ± 0.0	91.7 ± 0.4	195.3 ± 0.3

^1^ CN: cetane number; ^2^ SV: saponification value; ^3^ IV: iodine value; ^4^ CFPP: cold filter plugging point; ^5^ LCSF: long-chain saturated factor; ^6^ TFA: total fatty acid.

**Table 4:** Quality detection of the prepared SCC sample.

	E_1 cm_^1%^_405 nm_^3^	E_405_/E_630_^2^	pH	Yield (per 100 g Biomass)	Purity (%)
Prepared SCC ^1^ sample	59.0 ± 0.2	3.63 ± 0.09	8.25 ± 0.01	1.88 ± 0.04	10.2 ± 0.1
SCC standard (GB26406-2011 ^2^)	≥568	3.2~4.0	9.0~10.7	/	/

^1^ SCC: sodium copper chlorophyllin; ^2^ GB26406-2011: the standard of SCC in China; ^3^ E_1 cm_^1%^_405 nm_ represented extinction coefficient.

## Supplementary Materials

The following is available online at www.mdpi.com/1660-3397/14/9/162/s1, Figure S1: Biomass treated with saponification reagent (ethanol-NaOH).

## Abbreviations

The following abbreviations are used in this manuscript:

CN	cetane number
CFPP	cold filter plugging point
DW	dry weight
EU	Europe Union
FA	fatty acid
FDA	Food and Drug Administration
GLs	glycolipids
IV	iodine value
LCSF	long-chain saturated factor
MUFAs	mono-unsaturated fatty acids
NLs	neutral lipids
PLs	phospholipids
PUFAs	ploy-unsaturated fatty acids
SCC	sodium copper chlorophyllin
SEM	scanning electron microscope
SFAs	saturated fatty acids
SMC	sodium magnesium chlorophyllin
SV	saponification value
TEM	transmission electron microscope

## References

[1] Hu Q., Sommerfeld M., Jarvis E., Ghirardi M., Posewitz M., Seibert M., Darzins A. (2008). Microalgal triacylglycerols as feedstocks for biofuel production: Perspectives and advances. Plant J..

[2] Mata T.M., Martins A.A., Caetano N.S. (2010). Microalgae for biodiesel production and other applications: A review. Renew. Sustain. Energy Rev..

[3] Ramluckan K., Moodley K.G., Bux F. (2014). An evaluation of the efficacy of using selected solvents for the extraction of lipids from algal biomass by the soxhlet extraction method. Fuel.

[4] Garcia A.L. (2013). Algae Biorefinery: An Experimental Study on Liquid Fuels Production and Nutrients Recycling. Ph.D. Thesis.

[5] Hosikian A., Lim S., Halim R., Danquah M.K. (2010). Chlorophyll extraction from microalgae: A review on the process engineering aspects. Int. J. Chem. Eng..

[6] Aronoff S., Pirson A. (1960). The chemistry of chlorophyll. Die CO_2_-Assimilation/The Assimilation of Carbon Dioxide.

[7] Kulkarni M.G., Dalai A.K., Bakhshi N.N. (2006). Utilization of green seed canola oil for biodiesel production. J. Chem. Technol. Biotechnol..

[8] Diosady L.L. (2005). Chlorophyll removal from edible oils. Int. J. Appl. Sci. Eng..

[9] Issariyakul T., Dalai A.K. (2010). Biodiesel Production from Greenseed Canola Oil. Energy Fuels.

[10] Levadoux W.L., Kalmokoff M.L., Pickard M.D., GrootWassink J.W.D. (1987). Pigment removal from canola oil using chlorophyllase. J. Am. Oil Chem. Soc..

[11] Park J.-Y., Choi S.-A., Jeong M.-J., Nam B., Oh Y.-K., Lee J.-S. (2014). Changes in fatty acid composition of *Chlorella vulgaris* by hypochlorous acid. Bioresour. Technol..

[12] Baroi C., Dalai A.K. (2013). Simultaneous esterification, transesterification and chlorophyll removal from green seed canola oil using solid acid catalysts. Catal. Today.

[13] Bahmaei M., sadat Sabbaghian E., Farzadkish E. (2005). Development of a method for chlorophyll removal from canola oil using mineral acids. J. Am. Oil Chem. Soc..

[14] Ghazani S., Marangoni A. (2013). Minor components in canola oil and effects of refining on these constituents: A review. J Am. Oil Chem. Soc..

[15] Przybylski R., Mag T., Eskin N.A.M., Mc Donald B.E., Shahidi F. (2005). Canola Oil. Bailey’s Industrial Oil and Fat Products.

[16] Christaki E., Florou-Paneri P., Bonos E. (2011). Microalgae: A novel ingredient in nutrition. Int. J. Food Sci. Nutr..

[17] Sathish A., Sims R.C. (2012). Biodiesel from mixed culture algae via a wet lipid extraction procedure. Bioresour. Technol..

[18] Chen L., Liu T., Zhang W., Chen X., Wang J. (2012). Biodiesel production from algae oil high in free fatty acids by two-step catalytic conversion. Bioresour. Technol..

[19] Ferruzzi M.G., Blakeslee J. (2007). Digestion, absorption, and cancer preventative activity of dietary chlorophyll derivatives. Nutr. Res..

[20] Rodriguez-Amaya D.B. (2001). A Guide to Carotenoid Analysis in Foods.

[21] Yuan J.-P., Chen F. (1998). Chromatographic Separation and Purification of *trans*-Astaxanthin from the Extracts of *Haematococcus pluvialis*. J. Agric. Food Chem..

[22] Granado F., Olmedilla B., Gil-Martinez E., Blanco I. (2001). A Fast, Reliable and Low-cost Saponification Protocol for Analysis of Carotenoids in Vegetables. J. Food Compos. Anal..

[23] Pasquet V., Chérouvrier J.-R., Farhat F., Thiéry V., Piot J.-M., Bérard J.-B., Kaas R., Serive B., Patrice T., Cadoret J.-P. (2011). Study on the microalgal pigments extraction process: Performance of microwave assisted extraction. Process Biochem..

[24] Burja A.M., Armenta R.E., Radianingtyas H., Barrow C.J. (2007). Evaluation of fatty acid extraction methods for *Thraustochytrium* sp. ONC-T18. J. Agric. Food Chem..

[25] Kobayashi T., Tanabe I., Obayashi A. (1974). On the properties of the starch granules from unicellular green algae. Agric. Biol. Chem..

[26] Buléon A., Colonna P., Planchot V., Ball S. (1998). Starch granules: Structure and biosynthesis. Int. J. Biol. Macromol..

[27] Fernández-Reiriz M.J., Perez-Camacho A., Ferreiro M.J., Blanco J., Planas M., Campos M.J., Labarta U. (1989). Biomass production and variation in the biochemical profile (total protein, carbohydrates, RNA, lipids and fatty acids) of seven species of marine microalgae. Aquaculture.

[28] Rausch T. (1981). The estimation of micro-algal protein content and its meaning to the evaluation of algal biomass I. Comparison of methods for extracting protein. Hydrobiologia.

[29] Stansell G., Gray V., Sym S. (2012). Microalgal fatty acid composition: Implications for biodiesel quality. J. Appl. Phycol..

[30] Carr R. (1978). Refining and degumming systems for edible fats and oils. J. Am. Oil Chem. Soc..

[31] Zhou X., Xia L., Ge H., Zhang D., Hu C. (2013). Feasibility of biodiesel production by microalgae *Chlorella* sp. (FACHB-1748) under outdoor conditions. Bioresour. Technol..

[32] Hanagata N., Dubinsky Z. (1999). Secondary carotenoid accumulation in *Scenedesmus komarekii* (Chlorophyceae, Chlorophyta). J. Phycol..

[33] Aydin M.E., Farag A.A.M., Abdel-Rafea M., Ammar A.H., Yakuphanoglu F. (2012). Device characterization of organic nanostructure based on sodium copper chlorophyllin (SCC). Synth. Met..

[34] Shi Y., Yan G., Li Y. (2009). Research on the extraction technology of chlorophyll from silkworm excrement. Anim. Husb. Feed Sci..

[35] Whittick A. (1981). Handbook of phycological methods. Developmental and cytological methods. Phycologia.

[36] Lichtenthaler H.K. (1987). Chlorophylls and carotenoids: Pigments of photosynthetic biomembranes. Methods Enzymol..

[37] Li T., Wan L., Li A., Zhang C. (2013). Responses in growth, lipid accumulation, and fatty acid composition of four oleaginous microalgae to different nitrogen sources and concentrations. Chin. J. Oceanol. Limnol..

[38] Khozin-Goldberg I., Shrestha P., Cohen Z. (2005). Mobilization of arachidonyl moieties from triacylglycerols into chloroplastic lipids following recovery from nitrogen starvation of the microalga *Parietochloris incisa*. BBA Mol. Cell Biol. Lipids.

[39] Dubois M., Gilles K.A., Hamilton J.K., Rebers P.A., Smith F. (1956). Colorimetric method for determination of sugars and related substances. Anal. Chem..

