# Supplementary material for: A Saponification Method for Chlorophyll Removal from Microalgae Biomass as Oil Feedstock
Source: Mar Drugs. 2016 Sep 7;14(9):162. doi: 10.3390/md14090162 (PMC5039533; doi:10.3390/md14090162)
Supplement: Supplementary file 1 [file marinedrugs-14-00162-s001.pdf]

Supplementary Materials: A Saponification Method for Chlorophyll Removal from Microalgae Biomass as Oil Feedstock

Tao Li, Jin Xu, Hualian Wu, Guanghua Wang, Shikun Dai, Jiewei Fan, Hui He and Wenzhou Xiang

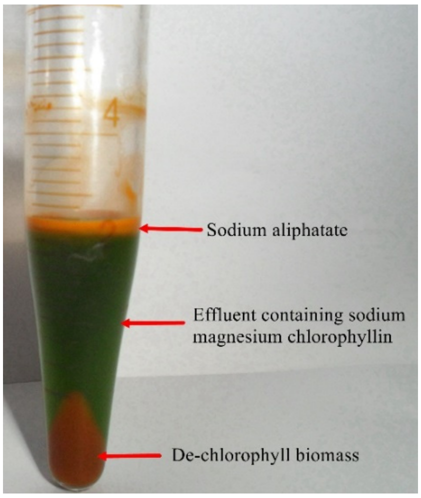

Figure S1. Biomass treated with saponification reagent (ethanol-NaOH).
